# Vitamin D Levels in Asymptomatic Children and Adolescents with Atopy during the COVID-19 Era

**DOI:** 10.3390/jpm11080712

**Published:** 2021-07-25

**Authors:** Gavriela Feketea, Vasiliki Vlacha, Georgios Tsiros, Panagiota Voila, Raluca Maria Pop, Ioana Corina Bocsan, Luminita Aurelia Stanciu, Mihnea Zdrenghea

**Affiliations:** 1 Department of Haematology, “Iuliu Hatieganu” University of Medicine and Pharmacy, 400337 Cluj-Napoca, Romania; gabychri@otenet.gr (G.F.); mzdrenghea@umfcluj.ro (M.Z.); 2 Hospital Unit of Amaliada, Department of Paediatrics, 27200 Amaliada, Greece; 3 Department of Paediatrics, Karamandanio Children’s Hospital, 26331 Patras, Greece; vasovlaha@gmail.com; 4 Department of Early Years Learning and Care, University of Ioannina, 26331 Ioannina, Greece; 5 Gastouni Health Centre, Department of Family Medicine, 27300 Gastouni, Greece; geotsiro@otenet.gr; 6 Private Medical Laboratory, Clinical Chemistry Department, 27200 Amaliada, Greece; zeta-med@otenet.gr; 7 Department of Pharmacology, Toxicology and Clinical Pharmacology, “Iuliu Hatieganu” University of Medicine and Pharmacy, 400337 Cluj-Napoca, Romania; raluca.pop@umfcluj.ro; 8 National Heart and Lung Institute, Imperial College London, London W2 1PG, UK; l.stanciu@imperial.ac.uk; 9 Ion Chiricuta Oncology Institute, Republicii Str., No. 34-36, 400010 Cluj-Napoca, Romania

**Keywords:** vitamin D, atopy, COVID-19

## Abstract

This study assessed vitamin D status in asymptomatic children and adolescents in Greece, with and without atopy, and possible changes during the coronavirus disease 2019 (COVID-19) pandemic. Serum levels of 25-hydroxy-vitamin D (25(OH)D) and total immunoglobulin E (IgE), and eosinophil count were measured in 340 asymptomatic children and adolescents (155 males, 185 females), mean age 8.6 ± 4.6 years, recruited over a period of 24 months (February 2019–January 2021). Atopy, defined by high level of IgE for age, was associated with vitamin D deficient status (*p* = 0.041). Subjects with and without atopy showed similar rates of insufficient and normal levels of 25(OH)D. The median level of 25(OH)D was significantly higher in subjects recruited during the pandemic, when home confinement rules were observed, than before the pandemic, and significantly more children had normal levels of 25(OH)D (*p* < 0.001), but no differences were noticed for IgE levels or eosinophil count. These results support a link between vitamin D and allergic and infectious inflammations, and specifically the association of vitamin D deficiency with asymptomatic atopy, defined as increased IgE level for age.

## 1. Introduction

Atopy is defined as either a personal, familial, or in combination, tendency, to have an immunological response after exposure to allergens, that are usually proteins, leading to differentiation of T helper cells (Th2), synthesis of immunoglobulin E (IgE) antibodies and allergic inflammation [1,2]. Atopic status can be assessed by skin prick tests to aeroallergens, and serum levels of total and specific IgE [3]. A raised serum level of total IgE, despite certain well-established limitations, is included as a diagnostic marker for allergic disease [4]. Epidemiological studies have demonstrated association of various allergic diseases, like asthma and allergic rhinitis, and atopic dermatitis, with an increase in the serum level of total IgE [5]. A high serum level of IgE is a good predictor of atopy and could be considered a marker of allergic airway inflammation in children with allergic disease [6]. Atopy may be asymptomatic [7], and studies have demonstrated airway inflammation in subjects with atopy without clinical manifestations, raising the hypothesis of subclinical inflammation in subjects with asymptomatic atopy [8,9]. 

The importance of vitamin D in childhood represents a public health pursuit worldwide, especially in developed countries. Low blood levels of vitamin D have been found to be correlated with the development of, apart from skeletal pathology, extra-skeletal problems in childhood, including allergy and autoimmune disorders [10]. The evidence regarding the relationship of the level of 25 hydroxy vitamin D ((25(OH)D) in umbilical cord blood with the development of allergic disease in infancy and early childhood is conflicting [11]. Baïz and colleagues reported an inverse association of cord 25(OH)D level with the risk of transient early wheezing and atopic dermatitis by the age of 5 years, but no relationship respiratory allergies, like asthma and rhinitis [12]. Chiu and colleague demonstrated a relationship between low 25(OH)D level in cord blood and sensitization to milk proteins, but not with asthma, allergic rhinitis or atopic dermatitis in early childhood [13]. Although the optimal level of vitamin D for decreasing the risk of development, and the severity of childhood allergies is still unclear, recent studies reinforce the concept that low levels increase the risk of clinical manifestations of atopy, such as bronchial asthma and allergic rhinitis [14]. Current data on vitamin D effects in inflammation, either infectious or allergic, are still conflicting [14,15,16,17,18]. One experimental study showed that a vitamin D deficient status in early childhood does not affect airway hyperreactivity, but that it aggravates eosinophilic inflammation and the airway remodeling process [19]. Experimental observations, however, need confirmation in human studies, and to date, the findings in clinical trials have been controversial.

The main objective of the present study was to investigate the serum level of 25(OH)D in asymptomatic children and adolescents with atopy. The secondary objective was to evaluate to what degree, if any, the pandemic restrictions imposed for control of COVID-19 have had an impact on the vitamin D status in these groups.

## 2. Materials and Methods

A 2-year prospective, observational, horizontal study was conducted from February 2019 to January 2021 in the district of Amaliada, in the Peloponnese, in Greece. The study was reviewed and approved by the Hospital Ethics and Scientific Committee of Hospital Unit of Amaliada, and written informed consent was provided by the parents of all the study children. Children who were referred to our laboratory for blood testing were initially screened. Subsequently, healthy children with vitamin D and IgE measurement conducted simultaneously were included in the study. Those children visited the pediatric clinic for regular checkups. The laboratory tests of interest were added to their screening lab test (for anemia and iron deficiency). During the study period, white subjects of both genders were enrolled, aged from 1 to 18 years, with a body mass index (BMI) below the 85th percentile for age and sex and without any symptoms, including those of allergy. The subjects had no underlying illnesses and were taking no vitamin supplementations or any other medications. Exclusion criteria were the administration of vitamin D supplements within the previous 6 months, comorbidities that could affect vitamin D status, chronic health conditions, known atopy, conditions known to increase IgE levels (such as parasitosis), and high BMI (because of the association between obesity and vitamin D level). None of the children had symptoms of infection within 2 weeks before biological evaluation. 

Commonly, the term “atopic” is used to describe an “IgE-mediated” disorder [20]. The children with high serum IgE levels for their age were characterized as subjects with atopy. The normal levels of serum IgE for corresponding age were 0–1 y, <15 IU/mL; 2–5 y, <60 IU/mL; 6–9 y, <90 IU/mL; 10–15 y, <200 IU/mL; and over 15 y, <100 IU/mL.

If they had no current or past symptoms of any allergic disease, they were defined as having “asymptomatic atopy”, for comparison with the children without atopy. Criteria for the diagnosis of “asymptomatic atopy” included a free medical history and no clinical findings of any allergic manifestation (e.g., food allergy, atopic dermatitis, allergic rhinitis, and asthma).

Peripheral blood eosinophils were quantified in a venous blood sample, collected on ethylenediaminetetraacetic acid (EDTA), using an automated blood cell counter (XE 2100; Sysmex xs 1000 hematology analyzer, Norderstedt, Germany) and expressed as a percentage and absolute number. For the determination of 25(OH)D and IgE, the blood samples were centrifuged immediately after collection, and the serum was analyzed. Furthermore, 25(OH)D and total IgE were measured by electrochemiluminescence binding assay, used on a Cobas e-411 immunoassay analyzer. Calibration and quality control were performed according to the manufacturers’ recommendations. The laboratory followed the established procedures for corrective measures when the values fell outside the defined limits.

The study sample was divided into the following subgroups. According to age, the subjects were divided into 2 subgroups: infancy and early childhood (1–4 years), and middle childhood and adolescence (5–18 years). Based on the levels of 25(OH)D, 3 subgroups were defined: deficient vitamin D (25(OH)D < 20 ng/mL), insufficient vitamin D (25(OH)D = 20–30 ng/mL), and normal vitamin D (25(OH)D > 30 ng/mL). According to the serum level of total IgE, 2 subgroups were identified: children with atopy (IgE level increased for age) and children without atopy (IgE level normal for age).

### Statistical Analysis 

Statistical analysis was performed using the SPSS 22 software program. Data were labelled as nominal and continuous variables. The nominal variables were characterized as percentages and frequencies. Normal distribution for continuous variables was tested using the Kolmogorov–Smirnov test. Variables with normal distribution were characterized as mean and standard deviation (mean ± SD), and those with non-normal distribution as median and 25–75 percentiles. Comparisons between groups were performed using Mann–Whitney or chi-square tests, whenever appropriate. Spearman rho coefficient was used for examining correlation between variables. ROC curves were used to find a cut-off value for quantitative variables that could discriminate between subjects with or without atopy. The level of statistical significance was set at *p* < 0.05. 

## 3. Results

### 3.1. The Linkage between Vitamin D and Atopy

During the study period, 340 children and adolescents (155 males and 185 females), with a mean age of 8.6 ± 4.6 years, were recruited. The demographic characteristics, atopic status, and vitamin D status are shown in Table 1.

The sub-groups of children with and without atopy were of similar age, but atopy was more frequent in boys than in girls (*p* = 0.03). Atopy was associated with vitamin D deficiency (*p* = 0.041), while children with and without atopy showed similar percentages of insufficient and normal serum levels of 25(OH)D. A little less than one quarter of the study children had hypereosinophilia. Those with atopy had a significantly higher eosinophil count than those without atopy (*p* = 0.037) (Table 1). 

**Figure 1:**
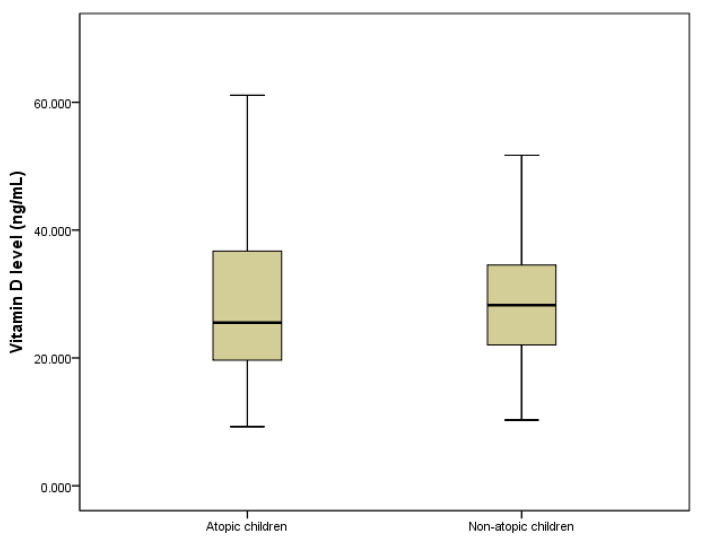
Box plot showing the levels of vitamin D (ng/mL) in children and adolescents with and without atopy (*n =* 340). Data are expressed as median, *p* < 0.064.

The median level of 25(OH)D was slightly higher in the children without atopy than in those with atopy, but the difference did not reach the level of statistical significance (*p* = 0.064) (Figure 1). The ROC curve for patients’ vitamin D levels was analyzed and the cut-off values were calculated for these parameters in relation with atopy presence. We calculated a cut-off value of 21.19 ng/mL that could discriminate between atopic and non-atopic patients (sensitivity 34.31 and specificity of 100%).

The eosinophil counts according to the age, sex, and vitamin D status of the study children are presented in Table 2.

More females than males had a normal eosinophil count, but the difference was not statistically significant. No differences in eosinophil count were found depending on the vitamin D status of the children, and there was no age difference.

The serum level of 25(OH)D was negatively correlated with the age of the children (R = −0.185, *p* = 0.001), but was not correlated with the eosinophil count. In children with atopy, total IgE was positively correlated with the eosinophil count (R = 0.172, *p* = 0.045), but not with 25(OH)D (R = 0.096, *p* = 0.172).

**Figure 2:**
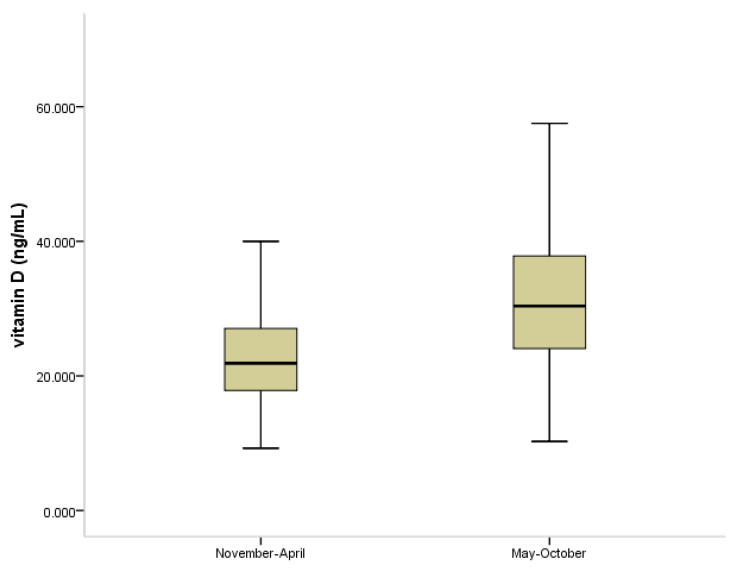
Box plot showing the level of vitamin D (25(OH)D, ng/mL) in children and adolescents with and without atopy (*n =* 340) according to season. Data are expressed as median, *p* < 0.001.

The level of 25(OH)D was higher in the children in whom the measurement was made during summer and autumn compared with winter and spring (*p* < 0.001) (Figure 2).

The level of total IgE and the eosinophil count showed no correlation with the season of measurement (*p* = 0.827, *p* = 0.658, respectively).

### 3.2. Vitamin D Status in COVID-19 Pandemia

**Figure 3:**
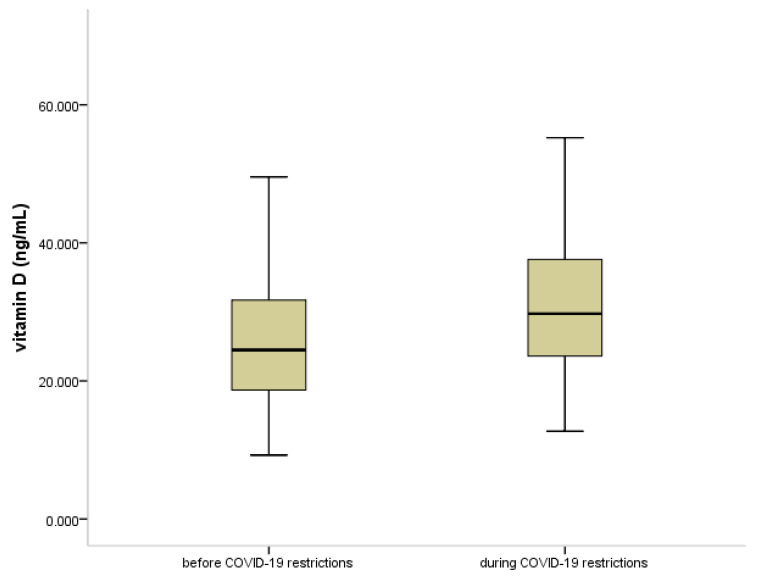
Box plot showing the level of vitamin D (25(OH)D, ng/mL) before and during COVID-19 restrictions. Data are expressed as median, *p* < 0.001.

The levels of 25(OH)D were also compared for the periods before (2019) and during (2020) the COVID-19 pandemic, in view of the restrictions applied. As shown in Figure 3, the levels of 25(OH)D were significantly higher in 2020 compared to 2019 (*p* < 0.001). Significantly more children and adolescents had normal levels of 25(OH)D during the pandemic (*p* < 0.001). The serum levels of IgE and the eosinophil count showed no significant differences between the two time periods (*p* = 0.956, *p* = 0.569, respectively).

Comparing the level of 25(OH)D in the two age groups prior to and during the COVID-19 pandemic, we found that during the pandemic, less children in both age groups were vitamin D deficient, and significantly more children were vitamin D sufficient (Table 3).

**Figure 4:**
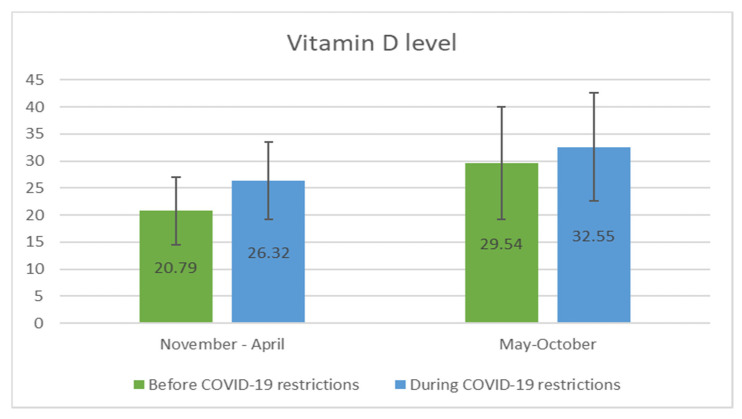
Box plot showing the level of vitamin D (25(OH)D, ng/mL) before and during COVID-19 restrictions, according to season of determination. The differences were statistically significant (*p* < 0.001).

Figure 4 shows the comparison of vitamin D status measured in the different seasons (November–April/May–October) prior to and during the pandemic. Significantly higher levels of 25(OH)D were noted in summer and autumn than in winter and the beginning of spring in both years of study (*p* < 0.001).

## 4. Discussion

The present study showed a possible linkage between asymptomatic atopy and vitamin D status in children and adolescents in Greece. The evidence to date concerning the exact role of vitamin D in the immunological mechanism of atopy and allergy is conflicting. Measurement of serum total IgE is a cost-effective test that can be used as a first-line screening tool to identify the status of atopy, and certain health insurance systems require a serum level of total IgE of greater than 100 IU/mL to justify testing for specific IgE [21]. Whether the presence of atopy, defined by a high IgE level for age, turn into clinically manifested allergy depends on a complex interplay of several factors, including the family history of atopy, allergen specific IgE or IgG, IgE against specific epitopes and serum factors that are still unidentified [22]. Vitamin D plays a central role in modulating the immune functions involved in occurrence of asthma and other allergic disorders, but an evident linkage between vitamin D status and allergy or asthma is contradictory. Most of the available experimental and epidemiological data indicate an association between low levels of 25(OH)D and the development of inflammation in atopy, asthma, and other allergic diseases [23,24,25]. A causal association has been proposed but has not been clearly demonstrated.

In our study, we found that a higher percentage of asymptomatic children and adolescents with atopy had vitamin D deficient status than their peers without atopy (Table 1), clearly indicating that vitamin D deficiency is more common in atopy. In asymptomatic children with atopy, allergic disease is not yet apparent, but allergic inflammation that is already present is likely to progress to allergic disease with various clinical manifestations [9]. Among subjects with asthma, the level of 25(OH)D is inversely associated with the degree of worsening of airflow limitation [26]. Hence, the finding of allergic inflammation in the children with asymptomatic atopy, despite the absence of clinical manifestations might be linked with their low 25(OH)D levels. Furthermore, 25(OH)D could be a biomarker for the presence of allergic inflammation, which has the potential to progress to clinical manifestations of allergic disease. In the case of already established allergic diseases (e.g., asthma, allergic rhinitis, or food allergy), the level of 25(OH)D has been clearly shown to be lower than that in healthy individuals [27,28]. The same relationship has been revealed for other causes of lung inflammation, such as cystic fibrosis [29].

The incidence of atopic manifestations is typically higher among boys [7], and our results are in accordance with this gender difference. As in other studies [30], our study showed an inverse relationship between 25(OH)D level and age in healthy children and adolescents.

In a recently published study from the same geographical region, a small percentage (1.3%) of children with an established food allergy needed administration of a dosage of vitamin D above the recommended age-specific daily requirements to maintain the 25(OH)D level within normal range [31]. Baek and colleagues suggested that low serum vitamin D levels are associated with sensitization to food allergens [32]. They have also shown that vitamin D status represents an independent risk factor for severe atopic dermatitis. Further evidence is needed to define the exact role of vitamin D in allergic inflammation and the progression to overt allergic disease. We found that serum 25(OH)D level was significantly reduced in children and adolescents with asymptomatic atopy (Figure 1), and thus 25(OH)D might be a potential biomarker for the existence of allergic inflammation in the asymptomatic phase of the disease.

In our study no association was observed between 25(OH)D median level and IgE level or eosinophil count. Other studies have also reported lack of association between 25(OH)D level and eosinophil count [26], but Brehm and colleagues reported an inverse relationship between circulating levels of vitamin D and specific allergy markers, like eosinophil count and IgE [33]. Other studies demonstrated no association between vitamin D and levels of IgE in asthmatic children [34]. Raised IgE levels denote the presence of atopy and allergic inflammation. In asymptomatic children with atopy, the absence of clinical manifestations of allergy does not exclude the presence of allergic inflammation; on the contrary, at the molecular level there is already an immune process of balancing towards the Th2 response. As previous research has shown that vitamin D is involved in Th2 inflammation [35,36], we would expect that in children with atopy, even in the absence of symptoms, the level of 25(OH)D would be low. Asymptomatic children who have low 25(OH)D levels could possibly benefit from correction of the 25(OH) level by vitamin D supplementation, but whether the normalization of the 25(OH)D level will affect the natural evolution of atopy in these children remains to be demonstrated by further studies.

The finding of a lower serum level of 25(OH)D in asymptomatic children with atopy leads us to believe that 25(OH)D is a biomarker of inflammation of the allergic type.

The increased levels of 25(OH)D observed during the pandemic could have several explanations, such as better dietary habits. One important factor could be the reduction in viral infections during that period, due to school closure and social distancing. The absence of infections, and therefore of viral inflammation, could result in higher levels of 25(OH)D in the COVID period compared with the same months of the pre-COVID era. This finding reinforces the idea that 25(OH)D is an index of inflammation in general. The prevalence of atopy, as defined by total IgE level increased for age, and the eosinophil count, did not change after the start of COVID-19 regulations.

The COVID-19 pandemic, apart from being a global public health crisis, created unique infectious epidemiological conditions, difficult to achieve in real life in other circumstances. Following the appearance of the first cases of COVID-19 and the introduction of the first measures to limit transmission, a decrease in respiratory viral infections has been observed in children [37]. During the H5N1 influenza virus epidemic, it was shown that the school closure during the holidays led to a 20–29% reduction in influenza transmission rate [38]. Shortly after the WHO declared the SARS-CoV-2 pandemic, the cases of influenza showed a sudden decrease, and the “flu season” ended earlier than usual. This trend, as the result of wearing facial masks and observing social distancing, has been demonstrated in several studies done in different regions of the world and for different respiratory viruses [39,40,41,42].

The connection between vitamin D and infections could be a two-way relationship, both cause and consequence. Viral respiratory infections may lead to a decrease in the vitamin D level, with longer and repeated infections resulting in a lower vitamin D level. Dogru and colleagues showed that in children with recurrent wheezing, the mean level of 25(OH)D was lower than in healthy control children and was negatively correlated with wheezing duration and numbers of wheezing episodes [43]. It is well established that the main cause of recurrent wheezing in childhood is successive viral respiratory infections. Some studies have shown no relationship or even a reverse relation [44]. An explanation for this could be that if the infections are not recurrent, or if the initial vitamin D status is sufficient, infection will not result in 25(OH)D reaching a deficient level. A recent meta-analysis conducted by Martineau et al. reported that vitamin D supplementation protected against acute respiratory infections, especially in patient’s vitamin D deficient status [45]. Mitchel, in a recent publication, questioned whether low vitamin D levels are a cause or consequence of respiratory infections [46]. During inflammation, either viral or atopic, there is an increased demand for vitamin D, which is important for local immunomodulation processes. Being practically a hormone [47], the level of vitamin D is regulated by feedback; hence, if the demand is so high that the body′s ability to produce it is exceeded, then the serum level of 25(OH)D will decrease. In such cases, it will be necessary to provide vitamin D supplement, and only in these cases, therefore, will supplementation favorably influence the evolution of the disease. This theory is supported by our study, where the absence of viral infections during the pandemic was associated with an increase in 25(OH)D levels (Figure 3), probably due to a decrease in demand for vitamin D. The presence of atopy, on the other hand, led to an increase in demand for vitamin D, resulting in a decrease in serum levels of 25(OH)D.

The UK National Institute for Health and Care Excellence (NICE) COVID-19 guidelines regarding vitamin D recommend that children aged 1–4 years should have a daily supplement containing 10 micrograms (400 units) of vitamin D throughout the year, while for those aged over 4 years this recommendation should be considered if they have little or no sunshine exposure or have dark skin [48]. In contrast with these guidelines, in Greece no such recommendation was issued by any regulatory body. In our study, as children who had taken vitamin D supplements in the previous months were excluded, the finding of high levels of 25(OH)D is not due to supplementation. In addition, the 25(OH)D levels observed in our study would not lead to such a dramatic decrease in infections observed in the COVID period, which most likely was a result of the COVID measures. We believe that the causal relationship is that less infection results in higher levels of 25(OH)D and not the reverse. In viral respiratory infections, vitamin D is used, locally, by immunomodulatory cells, [47,49] and its consumption results in a decrease in circulating 25(OH)D. Theoretically, the decrease would be greater when the infections are more serious or recurrent. In the COVID period, infections decreased dramatically, and consequently, less vitamin D was consumed, and thus the level of 25(OH)D was higher than in the previous year when common respiratory viral infections were prevalent among children during the winter.

Further investigation will be necessary to determine whether an enhanced inflammation with deficient/insufficient vitamin D status, even in the absence of clinical manifestations of atopy increases the risk of inflammation-related injury. The possible beneficial effect of vitamin D supplementation during asymptomatic allergy related inflammation also remains to be demonstrated.

### Strengths and Limitations of the Study

The main strengths of the study are that it presents the first real-life data investigating the link between the levels of 25(OH)D and total IgE in asymptomatic children and adolescents with atopy, and the differences in vitamin D status prior to and during the COVID-19 pandemic and its regulations. One limitation is the small sample size, and the lack of consecutive measurements. In addition, we were not able to investigate the possible influence of dietary habits on 25(OH)D level, time spent outdoors and indoors, and of sports activities, all of which may affect the level of 25(OH)D. In addition, we did not obtain a detailed history of the infections in the subjects, prior to and during the pandemic.

## 5. Conclusions

The study findings support a link between vitamin D status and different types of inflammation, allergic or infectious, as identified by total IgE status. Vitamin D deficiency was shown to be linked with atopy, defined as increased serum IgE level for age. Overall, the level of 25(OH)D was only slightly decreased in asymptomatic children and adolescents with atopy, compared with their healthy peers. During the pandemic, under the unprecedented circumstances consisting of almost complete absence of other infections, the serum levels of 25(OH)D were higher and fewer children were vitamin D deficient, probably due to a lower demand for vitamin D in immunomodulatory processes. Vitamin D deficient status may not be a risk factor for any types of inflammation, but rather a consequence of atopy or viral infection, through increased metabolic needs and its use by T and B lymphocytes, Th cells and macrophages in immunomodulation processes.

The level of 25(OH)D should be measured in asymptomatic children and adolescents with atopy, and if they are vitamin D deficient, the deficiency should be corrected by vitamin D supplementation, to change the natural history of allergic inflammation. This hypothesis will be of importance for developing preventive strategies for allergy in children.

## Figures and Tables

**Table 1:** Demographic characteristics and selected laboratory values in the study children with and without atopy *.

Parameter		Children with Atopy * (*n =* 137)	Children without Atopy (*n* = 203)	*p*
Age (years) ^^^		8 (5–13)	8 (5–11)	0.212
Sex	M	76 (55.5%)	79 (38.9%)	0.03
	F	61 (44.5%)	124 (61.1%)	
Vitamin D status	Deficient	37 (27%)	32 (15,8%)	0.041
	Insufficient	50 (36.5%)	85 (41.9%)	
	Normal	50 (36.5%)	86 (42.3%)	
Eosinophils	Hypereosinophilia **	35 (25.5%)	32 (15.8%)	0.037
	Normal	102 (74.5%)	171 (84.2%)	

* Atopy: based on serum level of IgE for age. ** Hypereosinophilia: absolute number of eosinophils above 500/μL. ^^^ Data are expressed as median; 25–75th percentile.

**Table 2:** Hypereosinophilia (HyperEo) according to the age, sex, and vitamin D status of the study children (*n* = 340).

Parameter		HyperEo * (*n* = 67)	Normal Eosinophil Count (*n* = 273)	*p*
Age ^^^ (years)		7 (5–11)	9 (5–12.5)	0.187
Sex	M	33 (49.3%)	122 (44.7%)	0.584
	F	34 (50.7%)	151 (55.3%)	
Vitamin D status	Deficient	14 (20.9%)	55 (20.1%)	0.723
	Insufficient	29 (43.3%)	106 (38.8)	
	Normal	24 (35.8%)	112 (41.1%)	

* Hypereosinophilia: absolute number of eosinophils > 500/μL. *^^^* Data are expressed as median; 25–75th percentile.

**Table 3:** Vitamin D deficiency before and during the COVID-19 pandemic, according to age.

Vitamin D Status	Age Group 1–4 Years (*n* = 76)			Age Group 5–18 Years (*n* = 264)		
	before Pandemic	during Pandemic	*p* Value	before Pandemic	during Pandemic	*p* Value
Deficient (25(OH)D < 20 ng/mL)	6 (16.2%)	2 (5.1%)	<0.05	44 (33.6%)	17 (12.8%)	<0.001
Insufficient (25(OH)D 20–30 ng/mL)	15 (40.5%)	12 (30.8%)		52 (39.7%)	56 (42.1%)	
Sufficient (25(OH)D > 30 ng/mL)	16 (43.3%)	25 (64.1%)		35 (26.7%)	60 (45.1%)	
Total	37	39		131	133	

## References

[1] Peebles R.S., Church M.K., Durham S.R., Holgate S.T., Church M.K., Broide D.H., Martinez F.D. (2012). Principles of allergy diagnosis. Allergy.

[2] Justiz Vaillant A.A., Modi P., Jan A. (2021). “Atopy”. Statpearls.

[3] Wüthrich B. (1999). What is atopy? Condition, disease or a syndrome?. Curr. Probl. Dermatol..

[4] Carosso A., Bugiani M., Migliore E., Antò J.M., Demarco R. (2006). Reference Values of Total Serum IgE and Their Significance in the Diagnosis of Allergy in Young European Adults. Int. Arch. Allergy Immunol..

[5] Zihlif M., Imraish A., Al-Rawashdeh B., Qteish A., Husami R., Husami R., Tahboub F., Jarrar Y., Lee S.-J. (2021). The Association of IgE Levels with *ADAM33* Genetic Polymorphisms among Asthmatic Patients. J. Pers. Med..

[6] Cardinale F., De Benedictis F.M., Muggeo V., Giordano P., Loffredo M.S., Iacoviello G., Armenio L. (2005). Exhaled nitric oxide, total serum IgE and allergic sensitization in childhood asthma and allergic rhinitis. Pediatr. Allergy Immunol..

[7] Thomsen S.F. (2015). Epidemiology and natural history of atopic diseases. Eur. Clin. Respir. J..

[8] Djukanovic R., Lai C.K., Wilson J.W., Britten K.M., Wilson S.J., Roche W.R., Howarth P.H., Holgate S.T. (1992). Bronchial mucosal manifestations of atopy: A comparison of markers of inflammation between atopic asthmatics, atopic nonasthmatics and healthy controls. Eur. Respir. J..

[9] Moody A., Fergusson W., Wells A., Bartley J., Kolbe J. (2000). Increased nitric oxide production in the respiratory tract in asymptomatic Pacific Islanders: An association with skin prick reactivity to house dust mite. J. Allergy Clin. Immunol..

[10] Antonucci R., Locci C., Clemente M.G., Chicconi E., Antonucci L. (2018). Vitamin D deficiency in childhood: Old lessons and current challenges. J. Pediatr. Endocrinol. Metab..

[11] Bountouvi E., Douros K., Papadopoulou A. (2017). Can Getting Enough Vitamin D during Pregnancy Reduce the Risk of Getting Asthma in Childhood?. Front. Pediatr..

[12] Baïz N., Dargent-Molina P., Wark J.D., Souberbielle J.-C., Annesi-Maesano I. (2014). Cord serum 25-hydroxyvitamin D and risk of early childhood transient wheezing and atopic dermatitis. J. Allergy Clin. Immunol..

[13] Chiu C.-Y., Yao T.-C., Chen S.-H., Tsai M.-H., Tu Y.-L., Hua M.-C., Yeh K.-W., Huang J.-L. (2014). Low cord blood vitamin D levels are associated with increased milk sensitization in early childhood. Pediatr. Allergy Immunol..

[14] Litonjua A.A. (2012). Vitamin D deficiency as a risk factor for childhood allergic disease and asthma. Curr. Opin. Allergy Clin. Immunol..

[15] Weiss S.T., Litonjua A.A. (2015). Vitamin D dosing for infectious and immune disorders. Thorax.

[16] Sabetta J.R., DePetrillo P., Cipriani R.J., Smardin J., Burns L.A., Landry M.L. (2010). Serum 25-Hydroxyvitamin D and the Incidence of Acute Viral Respiratory Tract Infections in Healthy Adults. PLoS ONE.

[17] Fabbri A., Infante M., Ricordi C. (2020). Editorial—Vitamin D status: A key modulator of innate immunity and natural defense from acute viral respiratory infections. Eur. Rev. Med. Pharmacol. Sci..

[18] Zdrenghea M.T., Makrinioti H., Bagacean C., Bush A., Johnston S., Stanciu L.A. (2017). Vitamin D modulation of innate immune responses to respiratory viral infections. Rev. Med. Virol..

[19] Vasiliou J., Lui S., Walker S.A., Chohan V., Xystrakis E., Bush A., Hawrylowicz C., Saglani S., Lloyd C.M. (2014). Vitamin D deficiency induces T h2 skewing and eosinophilia in neonatal allergic airways disease. Allergy.

[20] Johansson S.G.O., Hourihane J., Bousquet J., Bruijnzeel-Koomen C., Dreborg S., Haahtela T., Kowalski M.L., Mygind N., Ring J., Van Cauwenberge P. (2008). A revised nomenclature for allergy: An EAACI position statement from the EAACI nomenclature task force. Allergy.

[21] Chang Y.-C., Lee T.-J., Huang C.-C., Chang P.-H., Chen Y.-W., Fu C.-H. (2021). The Role of Phadiatop Tests and Total Immunoglobulin E Levels in Screening Aeroallergens: A Hospital-Based Cohort Study. J. Asthma Allergy.

[22] Bousquet J., Anto J.M., Bachert C., Bousquet P.J., Colombo P., Crameri R., Daeron M., Fokkens W., Leynaert B., Lahoz C. (2006). Factors responsible for differences between asymptomatic subjects and patients presenting an IgE sensitization to allergens. A GA2LEN project. Allergy.

[23] Nowak S., Wang H., Schmidt B., Jarvinen K.M. (2021). Vitamin D and iron status in children with food allergy. Ann. Allergy Asthma Immunol..

[24] Mirzakhani H., Al-Garawi A., Weiss S.T., Litonjua A.A. (2014). Vitamin D and the development of allergic disease: How important is it?. Clin. Exp. Allergy.

[25] Mirzakhani H., Carey V.J., Zeiger R., Bacharier L.B., O’Connor G.T., Schatz M.X., Laranjo N., Weiss S.T., Litonjua A.A. (2018). Impact of parental asthma, prenatal maternal asthma control, and vitamin D status on risk of asthma and recurrent wheeze in 3-year-old children. Clin. Exp. Allergy.

[26] Alyasin S., Momen T., Kashef S., Alipour A., Amin R. (2011). The Relationship Between Serum 25 Hydroxy Vitamin D Levels and Asthma in Children. Allergy Asthma Immunol. Res..

[27] Douros K., Loukou I., Boutopoulou B., Fouzas S. (2015). Does Vitamin D Deficiency Epidemic Parallel with Allergy and Asthma Epidemic?. Mini Rev. Med. Chem..

[28] Douros K., Boutopoulou B., Fouzas S., Loukou I. (2017). Asthma and Allergy “Epidemic” and the Role of Vitamin D Deficiency. Adv. Exp. Med. Biol..

[29] Loukou I., Moustaki M., Sardeli O., Plyta M., Douros K. (2020). Association of vitamin D status with lung function measurements in children and adolescents with cystic fibrosis. Pediatr. Pulmonol..

[30] Feketea G., Bocsan I., Tsiros G., Voila P., Stanciu L., Zdrenghea M. (2021). Vitamin D Status in Children in Greece and Its Relationship with Sunscreen Application. Children.

[31] Kalmpourtzidou A., Xinias I., Agakidis C., Mavroudi A., Mouselimis D., Tsarouchas A., Agakidou E., Karagiozoglou-Lampoudi T. (2021). Diet Quality: A Neglected Parameter in Children with Food Allergies. A Cross–Sectional Study. Front. Pediatr..

[32] Baek J.H., Shin Y.H., Chung I.H., Kim H.J., Yoo E.-G., Yoon J.W., Jee H.M., Chang Y.E., Han M.Y. (2014). The Link between Serum Vitamin D Level, Sensitization to Food Allergens, and the Severity of Atopic Dermatitis in Infancy. J. Pediatr..

[33] Brehm J.M., Celedón J.C., Soto-Quiros M.E., Avila L., Hunninghake G.M., Forno E., Laskey D., Sylvia J.S., Hollis B.W., Weiss S.T. (2009). Serum Vitamin D Levels and Markers of Severity of Childhood Asthma in Costa Rica. Am. J. Respir. Crit. Care Med..

[34] Thomas G.O., Tutar E., Tokuc G., Oktem S. (2019). 25-hydroxy Vitamin D Levels in Pediatric Asthma Patients and its Link with Asthma Severity. Cureus.

[35] Telcian A.G., Zdrenghea M.T., Edwards M.R., Laza-Stanca V., Mallia P., Johnston S.L., Stanciu L.A. (2017). Vitamin D increases the antiviral activity of bronchial epithelial cells in vitro. Antivir. Res..

[36] Han Y.-Y., Forno E., Boutaoui N., Canino G., Celedón J.C. (2018). Vitamin D insufficiency, TH2 cytokines, and allergy markers in Puerto Rican children with asthma. Ann. Allergy Asthma Immunol..

[37] Toelen J., Ritz N., de Winter J.P. (2021). Changes in pediatric infections during the COVID-19 pandemic: ‘a quarantrend for coronials’?. Eur. J. Nucl. Med. Mol. Imaging.

[38] Cauchemez S., Valleron A.-J., Boëlle P.-Y., Flahault A., Ferguson N.M. (2008). Estimating the impact of school closure on influenza transmission from Sentinel data. Nature.

[39] McDonnell T., Nicholson E., Conlon C., Barrett M., Cummins F., Hensey C., McAuliffe E. (2020). Assessing the Impact of COVID-19 Public Health Stages on Paediatric Emergency Attendance. Int. J. Environ. Res. Public Health.

[40] Polcwiartek L.B., Polcwiartek C., Andersen M.P., Østergaard L., Broccia M.D., Gislason G.H., Køber L., Torp-Pedersen C., Schou M., Fosbøl E. (2021). Consequences of coronavirus disease-2019 (COVID-19) lockdown on infection-related hospitalizations among the pediatric population in Denmark. Eur. J. Nucl. Med. Mol. Imaging.

[41] Poole S., Brendish N.J., Tanner A.R., Clark T.W. (2020). Physical distancing in schools for SARS-CoV-2 and the resurgence of rhinovirus. Lancet Respir. Med..

[42] Yeoh D.K., A Foley D., A Minney-Smith C., Martin A.C., Mace A.O., Sikazwe C.T., Le H., Levy A., Blyth C.C., Moore H.C. (2021). Impact of Coronavirus Disease 2019 Public Health Measures on Detections of Influenza and Respiratory Syncytial Virus in Children During the 2020 Australian Winter. Clin. Infect. Dis..

[43] Dogru M., Seren L. (2017). Serum 25-hydroxyvitamin D levels in children with recurrent wheezing and relation to the phenotypes and frequency of wheezing. Eur. Ann. Allergy Clin. Immunol..

[44] Feketea G., Bocsan C.I., Stanciu L.A., Buzoianu A.D., Zdrenghea M.T. (2020). The Role of Vitamin D Deficiency in Children With Recurrent Wheezing—Clinical Significance. Front. Pediatr..

[45] Martineau A.R., Jolliffe D.A., Hooper R.L., Greenberg L., Aloia J.F., Bergman P., Dubnov-Raz G., Esposito S., Ganmaa D., Ginde A.A. (2017). Vitamin D supplementation to prevent acute respiratory tract infections: Systematic review and meta-analysis of individual participant data. BMJ.

[46] Mitchell F. (2020). Vitamin-D and COVID-19: Do deficient risk a poorer outcome?. Lancet Diabetes Endocrinol..

[47] Sassi F., Tamone C., D’Amelio P. (2018). Vitamin D: Nutrient, Hormone, and Immunomodulator. Nutrients.

[48] NICE Guideline (2020). Covid-19 Rapid Guideline: Vitamin D. https://www.nice.org.uk/guidance/ng187/resources/covid19-rapid-guideline-vitamin-d-pdf-66142026720709.

[49] Norman A.W. (2008). From vitamin D to hormone D: Fundamentals of the vitamin D endocrine system essential for good health. Am. J. Clin. Nutr..

